# Opening paths to novel analgesics: the role of potassium channels in chronic pain

**DOI:** 10.1016/j.tins.2013.12.002

**Published:** 2014-03

**Authors:** Christoforos Tsantoulas, Stephen B. McMahon

**Affiliations:** 1 Department of Pharmacology, University of Cambridge, Cambridge CB2 1PD, UK; 2 Neurorestoration Group, Wolfson Centre for Age-Related Diseases, King's College London, London SE1 1UL, UK

**Keywords:** potassium channel, pain, dorsal root ganglia, pharmacotherapy

## Abstract

Chronic pain is associated with abnormal excitability of the somatosensory system and remains poorly treated in the clinic. Potassium (K^+^) channels are crucial determinants of neuronal activity throughout the nervous system. Opening of these channels facilitates a hyperpolarizing K^+^ efflux across the plasma membrane that counteracts inward ion conductance and therefore limits neuronal excitability. Accumulating research has highlighted a prominent involvement of K^+^ channels in nociceptive processing, particularly in determining peripheral hyperexcitability. We review salient findings from expression, pharmacological, and genetic studies that have untangled a hitherto undervalued contribution of K^+^ channels in maladaptive pain signaling. These emerging data provide a framework to explain enigmatic pain syndromes and to design novel pharmacological treatments for these debilitating states.

## Highlights

• Potassium (K^+^) channels are crucial determinants of neuronal excitability.
• Nerve injury or inflammation alters K^+^ channel activity in neurons of the pain pathway.
• These changes can render neurons hyperexcitable and cause chronic pain.
• Therapies targeting K^+^ channels may provide improved pain relief in these states.

## The problem of chronic pain

Chronic pain afflicts one in five adults in Europe and many diseases accompanied by pain are on the rise [1]. The diverse etiology of chronic pain encompasses trauma, metabolic or autoimmune disorders, infection, anti-retroviral treatment, and chemotherapy. Affected individuals typically report a combination of incapacitating sensory abnormalities, including spontaneous pain, hypersensitivity to stimulation, dysesthesias, and paresthesias. Despite significant progress, chronic pain remains refractory to treatment, with only one-third to two-thirds of patients reporting adequate (>50%) pain relief [1]. Moreover, our first-line drugs, non-steroidal anti-inflammatory agents (NSAIDS; e.g., aspirin) and opioids (e.g., morphine), are associated with adverse dose-limiting side-effects, dependence, and tolerance [2]. The lack of improved treatment reflects our incomplete understanding of the molecular pathophysiology underlying these pain states.

## Nociceptive pathways

Pain is usually triggered by the activity of specialized damage-sensing neurons innervating the limbs and torso, whose cell somata cluster paraspinally in the dorsal root ganglion (DRG). These pseudo-unipolar cells project axons that bifurcate into peripheral fibers innervating the skin, muscle, or other organs, and central fibers that synapse with second-order spinal cord neurons. A similar architecture is encountered in trigeminal ganglion neurons located on each side of the cranium, which transduce sensory information from the face. Based on anatomical, neurochemical, and functional attributes, sensory neurons are distinguished into small-diameter with unmyelinated C-fibers, medium-diameter with thinly myelinated Aδ-fibers, and large-diameter that principally give rise to heavily myelinated Aβ-fibers. Because of their ability to encode noxious mechanical, thermal, or chemical stimuli, C- and Aδ-fibers are considered the main nociceptive afferents signaling pain. Aβ-fibers innervating the skin or muscles are predominantly low-threshold mechanoreceptive afferents responding to light touch or pressure, although a proportion are also activated by high-threshold stimuli. Signals initiated at sensory endings are relayed to the dorsal horn of the spinal cord and subsequently the brain via spinal projection systems including the spinothalamic tract, where the information is evaluated and an appropriate response generated. Spinal transmission is not a passive process but rather involves regulatory spinal processing, such as facilitatory or inhibitory modulation by interneurons, astroglia, and descending pathways, which can robustly increase or decrease the output [3].

Under normal conditions, generation of action potentials (APs) in sensory nerves typically originates at their peripheral nerve endings in the presence of a suprathreshold stimulus activating specialized receptors. However, following nerve trauma, electrogenesis can occur spontaneously at the site of injury (neuroma), DRG cell body, or even mid-nerve [4]. Furthermore, inflammation and neuropathic lesions are linked to enhanced responsiveness to supra- or even subthreshold stimulation 5, 6, 7. This hyperexcitability is thought to be a major driver of pain and is ascribed to injury-induced reorganization of membrane ion channels, which are the principal determinants of AP generation and propagation. These maladaptive changes also have downstream effects at the spinal level; C-fiber activity can induce central sensitization, a state of heightened responsiveness of spinal cord neurons, such that innocuous input can now result in abnormally painful responses (e.g., tactile allodynia after Aβ-fiber stimulation) [8]. In addition, lesioned Aβ-fibers can acquire *de novo* nociceptive qualities that may also contribute to central sensitization [9].

Until recently the search for ion channel correlates of pathological excitability primarily focused on sodium and calcium channels. Unfortunately, despite significant discoveries in acute and inflammatory pain, no decisive involvement has been definitely established yet, particularly in neuropathic pain [10]. New evidence however suggests a previously unappreciated contribution of K^+^ channels in chronic pain processing, which we review here.

## K^+^ channels and pain signaling

K^+^ channels are the most populous, widely distributed, and diverse class of ion channels in neurons, governed by some 78 genes in humans [11]. Upon activation, K^+^ channels facilitate an extremely rapid transmembrane K^+^ efflux that can influence AP threshold, waveform and frequency. Because K^+^ channel opening repolarizes (or even hyperpolarizes) the neuronal membrane, this function can limit AP generation and firing rate.

**Figure 1:**
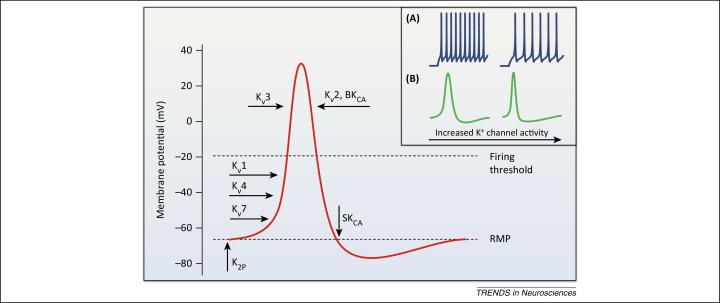
Potassium channel activation during action potential (AP) firing in sensory neurons. A depiction of the sequential engagement of different K^+^ channels during neuronal activity, and typical effects of K^+^ channel opening on AP waveform and frequency (inset). The resting membrane potential (RMP) is primarily stabilized by two-pore K^+^ (K_2P_) channels and K_v_7 background conductance, whereas K_ATP_ channels may also contribute in large neurons [95]. Basal excitability is also influenced by the opening of low-threshold K_v_1 and K_v_4 channels which filter out small depolarizations and therefore control the number of triggered APs. K_v_4 channels are normally inactivated at RMP and require prior hyperpolarization (achieved during AP generation) to remove this steady-state inactivation. Once activated, however, K_v_4 and other A-type channels may modulate firing threshold as well as repetitive spiking rate owing to their very fast kinetics [inset **(A)**] [47]. Following suprathreshold stimulation and initiation of an AP, high-threshold K_v_3 channels open to limit AP duration and ensure quick recovery of voltage-gated Na^+^ channels from inactivation [inset **(B)**]. K_v_2 channels are also high-threshold but with much slower activation and inactivation kinetics; they mainly contribute to the repolarizing/after-hyperpolarizing phases and are hence important for regulating interspike interval and conduction fidelity during sustained stimulation [inset (B)] [38]. Upon neuronal activity, Ca^2+^-activated K^+^ channels are engaged during repolarization (BK_CA_) and after-hyperpolarization (SK_CA_) to provide feedback inhibition at nerve terminals by restricting AP duration and thus neurotransmitter release [(inset (B)]. It is emphasized that this schematic is a simplified representation of most prominent K^+^ channel contributions to AP firing, based on *in vitro* assessment of recombinant counterparts. *In vivo*, however, the oligomeric composition, association with auxiliary proteins, post-translational modifications, and regulation by intracellular messengers can yield divergent biophysical properties. K^+^ channel opening will also have concurrent effects on the function of other ion channels, for instance by affecting their inactivation. For this reason the combined effect on firing behavior in a physiological context is often hard to predict. Abbreviations: BK_CA_, big conductance and SK_CA_, small conductance Ca^2+^-activated K^+^ channels.

Depending on the biophysical profile and precise subcellular localization in sensory neurons, K^+^ channel conduction is postulated to inhibit peripheral excitability by counteracting AP initiation at peripheral nerve terminals, reducing conduction fidelity across the axon, or limiting neurotransmitter release at central terminals (Figure 1). In addition, although normal sensory transduction does not rely on cell soma spiking, in chronic pain states K^+^ channels could provide a brake to the spontaneous activity developing in the DRG soma or other ectopic loci (e.g., the neuroma). Indeed, peripheral application of K^+^ channel openers on the cell body or terminals invariably decreases DRG excitability, whereas K^+^ channel blockers augment firing 5, 11, 12, 13. In the CNS, K^+^ channel opening could conceptually lead to enhanced nociception, for instance if the affected neuron participates in an inhibitory circuit. Nevertheless, the available data so far indicate that a variety of antinociceptive drugs mediate their action by directly opening spinal K^+^ channels [11].

Based on structural and physiological characteristics, K^+^ channels are organized into four distinct groups: voltage-gated, two-pore, calcium-activated, and inward rectifying, which we discuss in turn below.

## Voltage-gated K^+^ channels (K_v_)

The K_v_ superfamily is the most numerous among K^+^ channels, comprising of 40 genes in humans 14, 15, 16. They are further classified in 12 families of α subunits that can interact to form functional homo- or hetero-tetrameric channels. Members of K_v_1-K_v_4, K_v_7 and K_v_10-K_v_12 are pore-forming subunits, whereas K_v_5, K_v_6, K_v_8, and K_v_9 members do not form conducting channels unless associated with pore-forming subunits (Box 1). Channel tetramerization leads to tremendous functional diversity, further elevated by association with auxiliary β subunits, splice variants, and post-translational modifications.

The largely overlapping pharmacology in neurons suggests a spectrum of K_v_ currents rather than fixed groups, reflecting the variant heterotetrameric composition, functional redundancy within families, and complex regulation. The majority of K_v_ channels are delayed rectifiers, because they are activated slowly to counteract (rectify) depolarization. On the basis of biophysical properties and sensitivity to tetraethylammonium (TEA), α-dendrotoxin, 4-aminopyridine, and muscarinic agonists, K_v_ currents are broadly distinguished into sustained delayed rectifying (I_K_), transient slowly inactivating (I_D_), transient fast-inactivating (I_A_) and non-inactivating (I_M_) that, as their names suggest, exhibit different kinetics. Although this classification is an oversimplification, it has value as a starting point to examine the different K_v_ components in physiological systems.

These typical currents are also present in dorsal root and trigeminal ganglia neurons, whereas Gold and colleagues described six distinct K^+^ currents, three of which in small nociceptors 17, 18, 19, 20. Although it has been known for some time that nerve injury results in a dramatic decrease in K^+^ conductance of peripheral nerves that correlates with the emergence of hyperexcitability and pain behaviors, it was not until recently that specific subunits were linked to these changes [21].

K_v_1.1 and K_v_1.2 are delayed rectifiers activated by modest membrane depolarizations, and mainly contribute to the I_D_ current. In many CNS neurons, these channels are preferentially localized at the axon initial segment (AIS, the site of AP initiation in CNS neurons) where they regulate AP threshold and firing rates, as well as nerve terminals where they modulate neurotransmitter release by controlling AP invasion in axonal branches 22, 23. The dominant role of K_v_1 becomes apparent in type 1 episodic ataxia, where K_v_1.1 mutations drive excitability changes in the cerebellum that cause severe seizures and premature death [24].

In the peripheral nervous system (PNS), K_v_1.1 and K_v_1.2 are predominantly found in the soma and juxtaparanodes of medium-large DRG neurons, often in heterotetramers [25], and are largely decreased after axotomy 26, 27; this may contribute to the hyperexcitable phenotype. Indeed, K_v_1.1 loss-of-function results in reduced firing thresholds, attenuated mechanical and heat pain, and increased sensitivity in both phases of the formalin test 28, 29. By contrast, diminished K_v_1.2 activity contributes to mechanical and cold neuropathic pain by depolarizing the resting membrane potential (RMP), reducing threshold current, and augmenting firing rates in myelinated neurons [30]. Moreover, Hao *et al.* recently reported that K_v_1.1 tetramers form a bona fide mechanosensor that acts as an excitability brake in Aβ-mechanoreceptors of mouse DRG, with a minor contribution of K_v_1.2 [31]. Interestingly, this mechanosensitive current was also detected in some high-threshold C-mechano-nociceptors (C-HTMRs). Although the literature highlights predominant K_v_1.1 expression in myelinated neurons, the authors confirmed the presence of K_v_1.1 subunits in a subpopulation of capsaicin-insensitive small neurons and C-fiber terminals in the skin using a monoclonal antibody [30]. This pattern may correspond to the occasional expression Rasband *et al.* documented in small DRG neurons from rat [25]. Although species differences may account for the discrepancy (and multiple species variations are recognized), other studies implementing molecular, immunohistological, and electrophysiological techniques have also indicated presence of K_v_1.1 subunits in rat small sensory neurons 26, 29, 32, 33. Intriguingly, an accumulating body of research indicates that some human neuropathic pain syndromes are caused by production of autoimmune antibodies against K_v_1 subunits that disrupt normal A- or C-fiber function (Box 1).

Box 1 **Pain syndromes associated with autoimmune Kv antibodies**

Compelling evidence suggests that several neurological disorders linked to peripheral hyperexcitability and pain of a neuropathic nature, such as neuromyotonia (NMT) or Morvan's and cramp fasciculation syndromes, may be caused by erroneous K_v_ function due to host production of autoantibodies. K_v_ antibodies are detected in approximately 40% of NMT patients [111], and when transferred to mouse cells they cause reduction of K^+^ currents, DRG hyperexcitability and other signs of the disease [112]. In agreement with an autoimmune etiology, immunomodulatory therapy can improve function and symptoms [113]. Interestingly, these conditions can also arise owing to autoantibodies against proteins of the functional K_v_ complexes, such as Caspr2 or LGI1 [114]. Thus Caspr2 dysfunction may affect K_v_1 assembly at juxtaparanodes, whereas altered K_v_1 association with LGI1 in presynaptic C-fiber complexes could explain symptoms such as heat hyperalgesia. Although still in its infancy, the concept of K_v_ complex autoimmunity is an exciting development that may explain idiosyncratic pain in the absence of injury (e.g., fibromyalgia) or other presently enigmatic congenital pain states.

Most of our knowledge on K_v_2 comes from CNS studies, where K_v_2.1 and K_v_2.2 conduct the majority of delayed rectified I_K_ current in several neuron subtypes 15, 34. K_v_2 channels are activated slowly after significant depolarization, therefore their opening primarily influences membrane repolarization and inter-spike hyperpolarization during AP firing [15]. Importantly, because K_v_2 feature characteristically slow activation and inactivation, the progressive channel recruitment during sustained activity can have a cumulative limiting effect on firing rates. The prominent CNS function of K_v_2 is substantiated by specific localization in dendrites and AIS where the channel can exert intricate control over somal AP invasion and back-propagation [35]. Other interesting features of K_v_2 are the phosphorylation-dependent regulation by neuronal activity, which can fine-tune excitability of CNS neurons by altering the channel membrane distribution and biophysical properties [36], as well as their modulation by several silent K_v_ subunits [37].

Despite the pivotal K_v_2 role in shaping CNS signaling, an involvement in chronic pain was only recently uncovered. K_v_2 subunits are present in small nociceptors (where K_v_2.1 conducts the majority of I_K_ [34]) but are also abundantly expressed in myelinated DRG neurons [38]. Transcript and protein K_v_2 levels are downregulated by traumatic nerve injury, and this could augment firing by limiting the K_v_2 inhibitory effect on spike frequency 26, 27, 38. Indeed, application of a K_v_2 blocker on *ex vivo* DRG preparations promotes myelinated neuron hyperexcitability by increasing conduction fidelity to the cell soma during repetitive stimulation [38]. It is possible that particular subcellular K_v_2 localization forms the basis of an important filtering capacity (for instance by controlling AP traffic through the T-junction [39]), similarly to somatodendritic K_v_2 filtering of somatic input in the CNS. Finally, a role in supraspinal pain pathways has also been demonstrated; cortical expression of K_v_2.2 is reduced in oxaliplatin-induced neuropathy, and reproducing this *in vivo* results in marked cold and mechanical hypersensitivity [40].

All K_v_3 channels are high-threshold and are typically encountered in fast-spiking neurons where they facilitate AP repolarization and hence dictate AP duration, but without affecting AP threshold or interspike interval [41]. K_v_3.1 and K_v_3.2 are delayed rectifiers contributing a small fraction (20%) of I_K_ in small nociceptors, with a possible participation of K_v_3.3 heterotetramers [42]. The K_v_3.4 member almost certainly underlies the TEA-sensitive high-threshold transient current detected in nociceptors by Gold *et al*. (1996). This rapid K_v_3.4 current accelerates nociceptor repolarization, an effect that restricts Ca^2+^-dependent neurotransmitter release at central nerve endings, where K_v_3.4 is localized 41, 43. Hence, the mechanical hypersensitivity reported after K_v_3.4 antisense treatment can be explained by AP broadening and therefore increased neurotransmission [44], although a loss of protein kinase C (PKC)-dependent modulation has also been suggested [43]. It is noted that although APs only spend a brief time at voltages capable of activating K_v_3 channels, this restriction may be overcome by enhanced K_v_3 densities at sites of action [41]. In addition, in native channels the activation threshold of K_v_3.4 could be hyperpolarized following association with other proteins. For instance, heterotetramers of K_v_3.4 with the delayed rectifiers K_v_3.1 or K_v_3.2 are activated at –30 mV [45], whereas K_v_3.4 association with the auxiliary MinK-related peptide 2 (MiRP2) yields subthreshold currents in skeletal muscle [46].

In addition to K_v_3.4, K_v_4 members and K_v_1.4 also give rise to transient A-currents (I_A_) that inactivate rapidly [15]. In contrast to K_v_3.4, these A-channels are activated by small depolarizations, and their function in DRG can limit AP threshold, duration, and firing frequency [47]. Two low-threshold I_A_ are detected in DRG neurons [17]; although K_v_1.4 might contribute to the low-threshold I_A_ in small DRG neurons, the fast voltage-dependent recovery from inactivation suggests the presence of K_v_4 channels [25]. Therefore the low-threshold component may be predominantly mediated by K_v_4.1, and the somatically confined K_v_4.3 [48], because K_v_4.2 is either absent or expressed at very low levels 27, 48. Consistent with a role in nociceptive pathways, A-type subunit expression and currents in the DRG are found to be reduced in a variety of pain models 21, 25, 44, 49. Mimicking K_v_4.3 downregulation via intrathecal antisense is sufficient to induce mechanical hypersensitivity in naïve rats, presumably via reducing firing thresholds in a subset of Mrgprd (Mas-related G protein-coupled receptor D) neurons [44]. A-type blockers or short interfering RNA (siRNA) treatment can also diminish the analgesia by diclofenac in bone cancer [49], although K^+^ channel-related antinociception by this drug may be principally conferred via direct opening of other voltage-gated (e.g., K_v_7 [50]), ATP-sensitive, or Ca^2+^-activated channels [51]. Finally, despite its negligible involvement in DRG excitability, K_v_4.2 can strongly modulate pain plasticity in dorsal horn neurons; thus K_v_4.2-null mice exhibit quicker mechanical pain resolution following nerve injury, as well as loss of extracellular signal-regulated kinase (ERK)-dependent sensitization in inflammatory models [52]. Although a few K_v_4 activators are available (e.g., NS-5806 and KW-7158), the pacemaking activity of K_v_4 channels in cardiac tissue is limiting for systemic applications.

K_v_7 channels open near RMP and underlie the low-threshold, non-inactivating M-current (I_M_) [20]. I_M_ serves as a native ‘voltage clamp’ that stabilizes RMP and regulates AP threshold and accommodation within AP trains, affording it a central role in modulation of neuronal excitability. Accordingly, mutations in the human K_v_7.2/K_v_7.3 encoding genes cause benign familial neonatal epilepsy due to excessive excitability in distal motor axons [20]. In the DRG, I_M_ mediated by K_v_7.2, K_v_7.3 and K_v_7.5 oligomers is the dominant subthreshold K^+^ current in small neurons and a significant component in larger neurons (together with K_v_1.1/K_v_1.2) [12]. K_v_7.2 and K_v_7.3 are enriched in nociceptor AIS and terminals (but see [53]) and in nodes of myelinated fibers, in contrast to the majority of K_v_ channels which occupy paranodes or juxtaparanodes 12, 53. K_v_7.2 and associated currents are reduced in DRG following neuropathic lesions, although the delayed onset of downregulation suggests a link to the maintenance rather than initiation of pain [54]; nevertheless, enhancement of residual I_M_ can reverse pain behaviors [55]. Reduced K_v_7 function is also involved in inflammatory pain, where I_M_ inhibition occurs via protease-activated receptor 2 (PAR2) activation and phospholipase C (PLC)-induced depletion of phosphatidylinositol-4,5-bisphosphate (PIP_2_), inositol trisphosphate (IP_3_)-mediated Ca^2+^ augmentation, or a combination of both [12]. Consistent with this, PLC activation by bradykinin results in Ca^2+^ release which inhibits I_M_, thus allowing Ca^2+^-activated Cl^−^ channels to amplify depolarizing input and trigger spontaneous firing [56]. The general purpose anti-inflammatory diclofenac has also been shown to directly activate K_v_7.2/K_v_7.3 channels [50].

The anticonvulsant retigabine, the most advanced K_v_ modulator, reduces excitability of animal 12, 57, 58 and human [59] axotomized nociceptive fibers by enhancing I_M_ via a hyperpolarizing shift in K_v_7.2/K_v_7.3 activation. Accordingly, both retigabine and its structural analogue flupirtine (used as an analgesic in Europe since 1984) are antinociceptive in a variety of inflammatory and neuropathic pain paradigms through both central and peripheral mechanisms 12, 60, 61, 62. Although retigabine failed to produce analgesia in a recent clinical trial of post-herpetic neuralgia, flupirtine is currently in Phase II trials for fibromyalgia pain. New activators such as the K_v_7.2/K_v_7.3-selective ICA-27243 are in the pharmaceutical pipeline because retigabine and flupirtine do not show strong selectivity among K_v_7 subunits and can additionally cause side-effects by interacting with other targets such as GABA receptors [63].

## Two-pore K^+^ (K_2P_) channels

K_2P_ have emerged as promising candidates for pain modulation owing to their cell type-specific expression and lower inter-family sequence identity. They are unique among K^+^ channels in that they contain two pore domains and co-assemble as dimers rather than tetramers. Under physiological conditions K_2P_ generate hyperpolarizing leak currents that stabilize cells below firing threshold, and disrupting this constitutive conductance results in depolarization and increased excitability [64]. Sensory neurons express many of the 15 members of the K_2P_ superfamily, including TWIK1 (two-pore weak inwardly rectifying K^+^ channel), the TWIK-related (TR) channels TRESK, TREK1, and TRAAK, as well as TASK1 (acid-sensitive K^+^ channel), and marked reductions have been documented in pain states 65, 66. The importance of K_2P_ in pain is highlighted by the discovery of a human K^+^ channelopathy; thus, familial migraine with aura is associated with a dominant-negative mutation in TRESK, a subunit strongly expressed in human trigeminal and dorsal root ganglia [67]. This fits well with the fact that migraine is associated with secretion of neuropeptides such as calcitonin gene-related peptide (CGRP) and substance P by meningeal nociceptors of the trigeminal ganglia, which may lead to sensitization. In a traumatic injury context, TRESK expression is decreased by axotomy, whereas pharmacological or siRNA inhibition induces C-fiber hyperexcitability and pain behaviors [68]. Contrarily, adenovirus-mediated spinal delivery of TRESK can reverse nerve injury-induced mechanical allodynia [69]. Another interesting but relatively unexplored member, TWIK1, is selectively expressed in medium-large DRG neurons and undergoes robust and persistent reductions by neuropathic injury [66].

A particularly noteworthy feature of K_2P_ channels is their activation by a wide range of physicochemical factors including volatile anesthetics [70]. For instance, TREK1 is coexpressed with TRPV1 in nociceptors and can be activated by heat, stretching or lipids; a corresponding current is recorded in small neurons from wild type, but not TREK1 knockout (KO) animals [71]. TREK1 KO animals also show increased sensitivity to heat and mechanical stimulation, suggesting that normal K_2P_ function counterbalances inward currents generated by TRPV1 and mechanosensitive Na^+^-permeable channels, respectively. Interestingly, TREK1 activity is decreased by inflammatory mediators such as prostaglandin E_2_ (PGE_2_) and lysophosphatidic acid, and TREK1-null mice develop more modest mechanical and thermal hyperalgesia during inflammation, presumably due to loss of this inhibition 71, 72. These data suggest that TREK1-modulating drugs may be useful in acute and inflammatory pain. Although TREK1 KOs show reduced cold pain after SNL, the precise involvement of this channel in neuropathic pain has not been thoroughly examined [71]. The member TRAAK is also mechano- and heat-sensitive, and simultaneous deletion of TREK1 and TRAAK has additive effects that may explain some of the mechano-hypersensitivity in colitis 73, 74. Furthermore, double TREK1/TRAAK KOs exhibit defects in acute cold pain processing, traced back to menthol-insensitive nociceptors [74]. Interestingly, oxaliplatin reduces TREK1 and TRAAK expression, and double KOs have modified cold pain responses in this model [75].

## Ca^2+^-activated K^+^ channels (K_CA_)

Opening of K_CA_ during neuronal firing hyperpolarizes the membrane and provides feedback inhibition that limits Ca^2+^ influx and excitability, making them powerful regulators of synaptic transmission at nerve terminals [11]. Based on their conductance, they are further divided into BK_CA_ (big conductance), IK_CA_ (intermediate conductance), and SK_CA_ (small conductance).

All K_CA_ are found in DRG and respond to increases in intracellular calcium, whereas BK_CA_ are also voltage-sensitive 76, 77. Big conductance K_CA_ are thought to influence excitability more prominently; illustrative of their significance in pain transduction is the recent finding of a functional coupling with TRPV1 (transient receptor potential cation channel, subfamily V, member 1) in nociceptors [78]. Blocking these channels with iberiotoxin reduces outward currents, prolongs AP duration, and increases firing rates in small-medium sensory neurons, with no effect on RMP, AP threshold, or AP amplitude [77]. Accordingly, axotomy decreases BK_CA_ expression and Ca^2+^-dependent post-spike after-hyperpolarization in small-medium DRG neurons [79]. Contrariwise, the specific BK_CA_ opener NS-1619 suppresses DRG neuron firing and can even antagonize the hyperexcitability evoked by A-channel block [77]. Interestingly, PGE_2_ and other inflammatory mediators reduce BK_CA_ channel activity in nociceptors 17, 76, 80 and BK_CA_ deletion in these neurons enhances inflammatory pain without affecting acute or neuropathic behaviours [133]. The BK_CA_ opener andolast is currently in Phase III trials as an anti-inflammatory for chronic obstructive pulmonary disease; it would be interesting to evaluate the antinociceptive properties of NS-1619 and andolast in chronic pain models.

Smaller conductance K_CA_ are detected in a mixture of human and rodent DRGs, and may also contribute to pain phenotypes 76, 81. In small neurons, SK_CA_ are downstream targets of NMDA receptor (NMDAR)-mediated Ca^2+^ influx because deleting the NR1 subunit in DRG induces hyperexcitability and pain hypersensitivity that can be reproduced by NMDAR antagonism or pharmacological SK_CA_ inhibition [82]. Although IK_CA_ expression in large neurons is decreased by nerve injury, SK_CA_ and IK_CA_ subunits in small neurons appear unaltered, suggesting that opening these channels may be a viable approach for chronic pain relief [76]. In line with this, the channel opener 1-ethyl-2-benzimidazolone (1-EBIO) reduces excitability in response to mechanical stimulation; however, the analgesic properties of such compounds remain to be robustly tested 11, 83.

K_CA_ also participate in central pain processing. Nerve injury leads to enhanced BK_CA_ expression in second-order neurons near the dorsal root entry zone, and activating these channels by intrathecal NS-1619 reverses pain hypersensitivity [79]. Conversely, K_CA_ blockers can antagonize the antinociceptive effects of muscarinic receptor agonists, gabapentin, and perhaps some NSAIDS [11].

## Inward rectifiers (K_ir_)

These channels are expressed mainly (but not exclusively) in supporting cells (Box 2) and can conduct atypical inward (rather than outward) K^+^ currents at depolarized membrane potentials. This buffering activity adds to glial K^+^ uptake through electrogenic Na^+^/K^+^ pumps to offset extracellular K^+^ accumulation during neuronal firing [84], thus preventing AP ‘short-circuiting’ and uncontrolled excitability changes [85]. They belong to one of seven families (K_ir_1-K_ir_7) and have a relatively simple structure with two transmembrane domains flanking the pore region [11]. Three families implicated in nociception are K_ir_3 (also known as G protein-regulated inward rectifiers K^+^ channels, GIRK), K_ir_2, and the ATP-sensitive channels (K_ATP_).

Box 2 **The involvement of glial K+ channels**

Accumulating evidence points towards a pain-modulating role of K^+^ channels in satellite glial cells (SGC). For example, an inward current is detected in SGC of the trigeminal ganglion. The member responsible appears to be K_ir_4.1 because no inward currents are detected in K_ir_4.1-null animals, alongside depolarized RMP and inhibition of K^+^ uptake 115, 116. Attenuation of K_ir_4.1 expression in SGC of the trigeminal ganglion by neuropathic lesions or antisense treatment results in spontaneous and evoked facial pain-like behaviors [117]. Similarly, CFA inflammation can suppress K_ir_4.1 expression and associated currents, leading to depolarized RMP [118]. In the spinal cord, nerve injury reduces expression of K_ir_6.1/SUR, whereas the K_ATP_ opener cromakalim relieves pain via regulation of astroglial gap junctions [119]. Put together, these studies suggest that heightened extracellular K^+^ due to impaired glial K^+^ homeostasis can cause downstream hyperexcitability of adjacent neurons, although a glutamate involvement is also plausible. Interestingly, K_ir_2.1, K_ir_2.3, and K_ir_6.2 are also present in Schwann cells at the nodes of peripheral nerves, and putative regulation by injury might regulate excitability of myelinated neurons in a similar fashion 94, 120. Another class of K^+^ channels involved in spinal SGC-dependent hyperexcitability are the microglia-expressed K_CA_, which participate in microglia activation and migration following injury [121]. Hence, intrathecal treatment with a BK_CA_ blocker inhibits P2X_4_ expression and BDNF synthesis in spinal microglia, and precludes injury-induced tactile allodynia. The analgesic effects of ketamine in neuropathic pain and inflammation may also be partly mediated via inhibition of microglia activation following K_CA_ current attenuation [121].

Neuronal GIRK channels are important determinants of spinal analgesia. As their name suggests they can interact with G proteins, an association thought to underlie the analgesic effects of opioids, endocannabinoids, and endogenous pain modulators [11]. Interestingly, enhanced GIRK1 phosphorylation in the dorsal horn following neuropathy or inflammation suggests reduced channel activity [86], whereas ‘pain risk’ GIRK2 alleles are associated with intensity of chronic back pain in humans [87]. Although no GIRK openers are currently available, their development could provide a viable alternative to opiates because this interaction may set in motion the same analgesic pathway without the unwanted side-effects of direct opioid activation 88, 89, 90. Furthermore, a recent study suggests that GIRK2 expressed in sensory neurons also contribute to peripheral opioid-mediated antinociception [134]. Finally, although normally expressed in low levels in the periphery, K_ir_2.1 channels could also be useful for therapeutic interventions; virus-mediated expression of K_ir_2.1 in DRG neurons can restore excitability following compression injury, and even preclude pain symptoms when applied pre-emptively [91].

K_ATP_ members are tetramers of K_ir_6.1 or K_ir_6.2 surrounded by four sulfonylurea receptor subunits (SUR1 or SUR2) [92]. These channels are inhibited by ATP but also modulated by ligands such as ADP, adenosine, NO, vasoactive intestinal polypeptide (VIP) and CGRP. K_ATP_ currents are generally thought to play a minor role in setting basal excitability of DRG neurons, where K_v_7 and K_2P_ conductances dominate [93]. However, a therapeutic potential in pathological conditions has been proposed. Thus, although K_ir_6.2 activity is reduced in large DRG neurons post-injury, the ability of K_ATP_ openers to hyperpolarize RMP is retained, which could be exploited for neuropathic pain treatments 94, 95. Similarly, the inhibition of K_v_7 activity in nociceptors during inflammation may also reveal analgesic roles for K_ATP_ channels. Indeed, the activators pinacidil and diazoxide reduce the hyperexcitability and pain induced by a range of peripheral inflammatory stimuli 51, 93, 96.

Finally, K_ATP_ opening in the CNS is linked to the antinociception produced by systemic treatment with morphine, NSAIDs, or even gabapentin 11, 97. Unfortunately, the involvement of K_ATP_ in modulation of cardiac rhythmicity, pancreatic insulin secretion, and intestinal function necessitates therapeutic strategies that selectively target the tissues of interest [92].

## How does nerve injury trigger K^+^ channel dysfunction?

**Figure 2:**
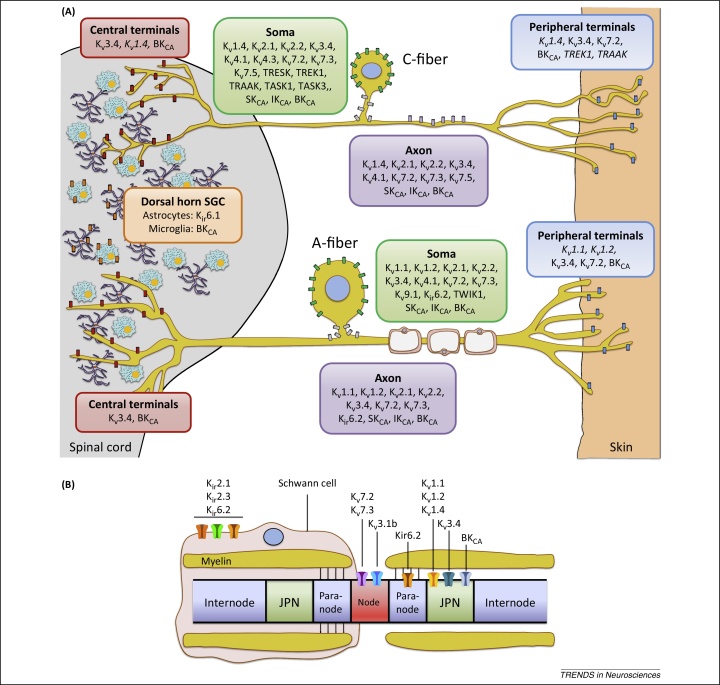
Expression and function of K^+^ channels in sensory neurons. **(A)** Subcellular localization of K^+^ channel subunits in unmyelinated (top) and myelinated (bottom) murine dorsal root ganglia (DRG) neurons. The panoply of K^+^ channels endows sensory neurons with a sophisticated machinery for the regulation of neuronal excitability. The depiction illustrated here is not absolute but rather reflects most prominent expression patterns in pain-relevant subpopulations, as reported in the literature. In addition, it is noted that K^+^ channel distribution patterns can vary tremendously between species, and validation against human data is currently very limited 59, 81. In the pain pathway, the TWIK-related (TR) channels TREK1 and TRAAK (and possibly TRESK) located at C-fiber terminals can counteract the activation of inward-conducting ion channels by pressure, heat or cold, whereas steady K_v_7 currents also stabilize RMP and regulate action potential (AP) threshold. In myelinated neurons, low-threshold K_v_1.1/K_v_1.2 heterotetramers appear to modulate acute and neuropathic pain modalities 28, 30, 31, whereas K_v_1.4 and K_v_4 members may exert similar roles in small nociceptors 25, 44. In addition, recent evidence suggest that K_v_1.1/K_v_1.2 may also function as mechanoreceptors in some C-fibers (not shown) [31]. Transmission of signals generated at the periphery is reliant on numerous axonal K^+^ channels, which influence the fidelity of AP conduction and therefore the fiber following frequency. Although normal sensory transduction is independent of spiking in the DRG soma, this can become a site of spontaneous firing in neuropathic conditions. In these scenarios, the activity of somal K^+^ channels may become an important regulator of excitability by influencing somal AP generation as well as propagation past the DRG T-junction. Potential candidates here are channels that preferentially localize at the soma or axon initial segments (in grey), such as K_v_4.3 in mechanosensitive C-fibers [44] or K_v_2/K_v_9.1 in A-fibers 38, 122. At the central terminals, Ca^2+^-activated channels BK_CA_ fine-tune activity and regulate neurotransmitter release in the spinal cord in response to calcium influx during AP firing. The high-threshold K_v_3.4 limits AP duration and thus may play a key role in synaptic transmission, whereas Kv1.2 may also regulate presynaptic terminal excitability [22]. Finally, pain processing can be influenced by K^+^ channels expressed by glial satellite cells (GSC) in the dorsal horn. Astrocyte-expressed K_ir_6.1 (and perhaps K_ir_3.1 [86]) buffers the extracellular K^+^ to maintain equilibrium potential during neuronal firing [119], and BK_CA_ conduction is involved in microgliosis following injury [121]. In addition, satellite cell-expressed K_ir_4.1 is involved in facial pain processing in the trigeminal ganglion (not shown). Subunits denoted in italics represent localizations that are indirectly implied by pharmacological profiling in DRG neurons, or by extrapolating on known localization in other neuronal types. For example, the K_v_1.1 and K_v_1.2 subunits are typically detected in dendrites and terminals of CNS neurons 22, 23, whereas TREK1 and TRAAK are axonally trafficked in sciatic nerves and are present at synaptic sites in cerebellar cultures [123]. **(B)** K^+^ channel composition of a myelinated DRG axon, illustrating nodes, paranodes, juxtaparanodes (JPN), internode segments, and a myelinating Schwann cell. The K_v_7.2 and K_v_7.3 subunits (together with a splice variant of K_v_3.1) are found in the nodes, and may therefore more prominently affect saltatory conduction under physiological conditions. Following axonal injury and demyelination, however, other channels such as the juxtaparanodal K_v_1 subunits may become exposed, leading to reduced conduction velocity and negative symptoms including sensory loss. In other cases, reduced axonal K^+^ channel function due to disrupted node organization (e.g., autoantibodies against K_v_ complex proteins) may induce peripheral hyperexcitability. Schwann cells also express inward rectifiers that regulate the node microenvironment during neuronal activity.

In the preceding paragraphs we reviewed studies describing distinct expression patterns of K^+^ channels involved in the peripheral and central processing of painful stimuli (Figure 2) as well as their extensive downregulation after nerve lesions (Table 1). The latter finding has implications for treatment because the analgesia produced by pharmacologically enhancing the remaining K^+^ activity may be of limited scope. In these cases, targeting upstream cascades that orchestrate K^+^ channel dysfunction could yield more efficacious treatments. For instance, it was recently reported that an injury-induced endogenous non-coding RNA attenuates K_v_1.2 expression, and blocking this pathway diminishes neuropathic pain [30]. Whether similar non-coding RNAs modulate the activity of other K^+^ channels is a question that warrants further investigation. Similarly, expression of K_v_7.2, K_v_4.3, and other ion channels in DRG is inhibited by the transcription factor REST (RE1-silencing transcription factor), which is induced by injury or inflammation 54, 98. Accordingly, blocking REST with antisense restores transcript levels and reverses some neuropathic pain symptoms [99].

**Table 1:** Summary of studies investigating the effect of altered K^+^ channel expression and function on acute, neuropathic, and inflammatory pain phenotypes

Superfamily	Subunit	Manipulation	Expression/excitability changes	Pain phenotype	Comments	Refs
Voltage-gated	K_v_1.1	KO transgenic	Loss of IK_mech_ currents ↓Mechanical threshold (HTM-C fibers) ↓Firing adaptation (Aβ-fibers)	↑Mechanical ↑Heat ↑Formalin	Reduced morphine antinociception	28, 31
		Morvan's syndrome Neuromyotonia	↑Peripheral excitability	↑Mechanical allodynia ↑Heat hyperalgesia	Autoantibodies to K_v_1 complexes; cause disease when transferred to cells; immunomodulatory therapy useful	111, 112, 113
	K_v_1.2	Nerve injury (SNL) K_v_1.2 knockdown	↓K^+^ current, ↓firing threshold ↑RMP, ↑firing rate	↑Mechanical ↑Cold	SNL induces K_v_1.2 antisense RNA; pre-emptive sense RNA alleviates pain	[30]
	Kv1.4	Nerve injury (SNT, SNL) Diabetic neuropathy Inflammation	↓K_v_1.4 expression	ND	–	25, 101, 124
	K_v_2.1	Nerve injury (SNL, SNT)	↓K_v_2.1 expression	ND	–	26, 38
	K_v_2.2	Nerve injury (SNL, CCI)	↓K_v_2.2 expression	ND	–	27, 38
		Oxaliplatin K_v_2.2 knockdown (cortex)	↓K_v_2.2 expression (cortex)	↑Mechanical ↑Cold	–	[40]
	K_v_3.4	Nerve injury (SNL) K_v_3.4 knockdown Diabetic neuropathy	↓K_v_3.4 expression ↑AP duration	↑Mechanical	PKC phosphorylation slows K_v_3.4 inactivation and decreases AP duration	43, 44, 101
	K_v_4.2	KO transgenic	DH neurons: ↑RMP, ↓firing threshold ↑Repetitive firing ↓I_A_ current,↑excitability	↑Mechanical, ↑heat ↓Formalin, ↓Carrageenan	Quicker resolution of mechanical allodynia after CCI; defects in central sensitization	[52]
	K_v_4.3	Nerve injury (SNL) Diabetic neuropathy K_v_4.3 knockdown	↓K_v_4.3 expression	↑Mechanical	REST antisense blocks K_v_4.3 downregulation	44, 99, 101
	K_v_7.2 K_v_7.3	Nerve injury (PSNL)	↓K_v_7.2 expression ↓I_M_ current	↑Mechanical ↑Heat	Perisciatic flupirtine reverses pain; antagonized by XE-991 blocker	[54]
		Bone cancer	↓I_M_ current, ↑excitability	↑Mechanical ↑Heat	–	[55]
		Retigabine (opener) after nerve injury or inflammation	↓Excitability (DRG, neuroma, DH neurons)	↓Mechanical (CCI, SNI, bone cancer) ↓Heat (bone cancer) ↓Cold (CCI) ↓Formalin, ↓Carrageenan ↓CFA ↓Visceral pain	Antinociception reversed by XE-991; no effect on intact fibers or acute pain	12, 55, 58, 60, 61, 62
	K_v_9.1	K_v_9.1 knockdown Nerve injury (SNL)	↓K_v_9.1 expression ↑SA, ↑firing, ↑after-discharge	↑Mechanical	Mediated through K_v_2	[122]
		Human SNPs	ND	↑Risk of phantom limb pain ↑Risk of chronic back pain ↑HIV neuropathy pain intensity	–	125, 126
Two-pore	TRESK	Human DN mutation	↓TRESK currents	↑Migraine pain	–	[67]
		Nerve injury (SNT) TRESK knockdown	↓TRESK expression	↑Mechanical	Trend for thermal pain	[68]
		TRESK overexpression after nerve injury (SNI)	↑TRESK expression	↓Mechanical allodynia	–	[69]
	TREK1	KO transgenic	↓TREK1 expression ↑AP firing	↑Mechanical, ↑heat ↓Inflammatory	Reduced cold hypersensitivity after SNL; TREK1 inhibited by PGE_2_ and cAMP	[71]
	TRAAK	KO transgenic	↓TRAAK expression	↑Mechanical, ↑heat	TREK1/TRAAK KO: ↑noxious cold pain but reduced cold sensitivity after oxaliplatin	74, 75
	TASK	Inflammation (CFA)	↓TASK1, TASK2, TASK3 expression	ND	–	[65]
	TWIK1	Nerve injury (SNI)	↓TWIK1 expression	ND	–	[66]
Inward rectifiers	K_ir_2.1	Nerve injury (CCD) plus K_ir_2.1 overexpression	↑K_ir_2.1 expression ↓RMP, ↑firing threshold, ↓SA	↓Mechanical hyperalgesia	Late treatment had no affect	[91]
	K_ir_3.1	Nerve injury (PSNL) Inflammation (formalin)	↑Phospho-K_ir_3.1 (SC)	ND	Predicted to increase excitability by decreasing K_ir_3.1 activity	[86]
	K_ir_3.2	Eight human SNPs	ND	↓Acute pain tolerance ↑Chronic back pain intensity	–	[87]
	K_ir_4.1	Nerve injury (CCI) K_ir_4.1 knockdown	↓K_ir_4.1 expression (SGC of TG)	↑Facial pain	–	[117]
		Inflammation (CFA)	↓K_ir_4.1 expression (SGC of TG) ↓K_ir_4.1 currents, ↑RMP (SGC of TG)	↑Facial pain	–	[118]
	K_ATP_	Nerve injury (CCI)	↓K_ir_6.1 expression (SGC of spinal cord)	↑Mechanical, ↑heat	Cromakalim (opener) reversed pain; antagonized by carbenoxolone	[119]
		Nerve injury (SNL)	↓K_ir_6.2 expression (DRG, Schwann cells) ↓K_ATP_ activity	↑Mechanical	Diazoxide (opener) or CAMKII activate residual K_ATP_ channels	94, 95
		Pinacidil and diazoxide (openers)	↓DRG excitability	↓Inflammatory ↓Mechanical, ↓thermal	Antagonized by K_ATP_ blocker glyburide	[93]
Calcium-activated	SK_CA_/IK_CA_	Apamin (blocker)	↑AP frequency, ↑excitatory transmission	↑Mechanical, ↑heat	NR1 KO in DRG has same effects	[82]
		Nerve injury (CCI) Inflammation (CFA)	No change in SK1–3 expression	ND	Targeting residual SK may be viable for pain treatment	[76]
		Nerve injury (avulsion)	↓IK1 expression	ND	IK1 was upregulated *in vitro* by NT-3	[81]
		1-EBIO (opener)	↓Excitability after mechanical stimulation	ND	Effects reversed by UCL-1848 (blocker)	[83]
	BK_CA_	Iberiotoxin (blocker)	↓BK_CA_ current ↑AP duration, ↑firing frequency	ND	NS-1619 (opener) decreases excitability	[77]
		Nerve injury (SNL)	DRG: ↓BK_CA_ expression SC: ↑BK_CA_ expression (DREZ)	↑Mechanical, ↑heat	Iberiotoxin reduces mechanical thresholds; BK_CA_ opener (NS-1619) reverses SNL pain	[79]
		Nerve injury (SNL)	↓BK_CA_ current (DRG) ↓ BKα1 mRNA (DRG)	ND	BDNF reduced BK_CA_ currents. Anti-BDNF reversed BK_CA_ reduction	[102]
		Inflammation (CFA)	↓BK_CA_ current (DRG)	ND	No change in BK_CA_ expression	[80]
		Charybdotoxin (blocker)	↓P2X_4_ and BDNF in microglia	↓Tactile allodynia	Mediated via inhibition of microglial activation	[121]
		KO transgenic	↓BK_CA_ expression (nociceptors)	↑Inflammatory	Acute and neuropathic pain unaffected	[133]

Unless stated otherwise, entries in expression and excitability refer to sensory neurons. Abbreviations: AGJ, astroglial gap junction; AP, action potential; CCD, chronic compression of the dorsal root ganglion; CFA, complete Freund's adjuvant; CCI, chronic constriction injury; DH, dorsal horn; DN, dominant-negative; DREZ, dorsal root entry zone; DRG, dorsal root ganglion; HTM, high-threshold mechanoreceptor; KO, knockout; ND, not determined; PSNL, partial sciatic nerve ligation; RMP, resting membrane potential; SA, spontaneous activity; SC, spinal cord; SGC, satellite glial cells; SNI, spared nerve injury; SNL, spinal nerve ligation; SNP, single-nucleotide polymorphism; SNT, sciatic nerve transection; TG, trigeminal ganglion.

Ion channel expression is typically controlled by carefully balanced neurotrophic support, which may become disrupted in pain pathology [100]. One of the most interesting messengers downstream of REST is brain-derived neurotrophic factor (BDNF), which has an established sensitizing role; in a diabetic neuropathy model, pre-emptive anti-BDNF treatment can reverse the I_A_ reduction in myelinated neurons [101]. The regulatory role of BDNF may be more general among K^+^ channels, because the injury-induced BK_CA_ downregulation in DRG can also be reversed by anti-BDNF [102]. There is also evidence that K_CA_ activity can be regulated by nerve growth factor (NGF), glial-derived neurotrophic factor (GDNF), and neurotrophin 3 (NT3) 81, 103, 104. Early research showed that NGF treatment can normalize axotomy-induced I_A_ and I_K_ reductions in DRG; however, an inhibitory effect on I_A_ and I_M_ may occur during inflammation 105, 106, 107. The exact influence of these growth factors and the responsive K^+^ channel subunits remains to be systematically tested and clarified.

## Concluding remarks

The exceptional abundance and breadth of function encountered in K^+^ channels has complicated efforts to untangle explicit roles in pain syndromes. Owing to advances in molecular, biochemical, electrophysiological, and genetic methods, however, we can now appreciate the involvement of specific subunits in maladaptive pain signaling after injury or inflammation. Nevertheless, there are many potential avenues of K^+^ involvement that have hardly been explored. It seems likely that unknown mutations in K^+^ channel genes might contribute to inherited pain syndromes. There are many ‘silent’ K^+^ channel subunits for which we have little idea of whether and how they might affect pain processing (Box 3). Auxiliary subunits can provide alternative substrates for pharmacological modulation; however, our understanding of these interactions in the PNS is also limited. In many chronic pain models an extensive dysregulation of several K^+^ channels is seen, and it is unknown whether a common epigenetic control exists.

Box 3 **Why so many silent and auxiliary Kv subunits?**

Emerging evidence suggests that the rich complement of modulatory K_v_ partners may have previously overlooked significance in fine-tuning neuronal activity. The silent K_v_9.1 subunit is selectively localized in myelinated DRG neurons, the principal source of spontaneous activity after nerve injury [5]. Injury-induced K_v_9.1 downregulation triggers spontaneous and evoked hyperexcitability as well as mechanical allodynia [122]. Intriguingly, a human K_v_9.1 polymorphism is associated with high risk of developing chronic back pain or persistent pain after amputation [125], whereas another study found a link with pain intensity in HIV neuropathy [126]. These effects are most likely mediated via a regulation of K_v_2 currents, which are modulated by K_v_9.1 *in vitro* [37]. Similar K_v_2 modification by the non-conducting K_v_5.1, K_v_6.1, K_v_6.3, K_v_8.1, and K_v_9.3 has been reported in heterologous systems and DRG neurons; it is therefore appealing to suggest that silent subunits might be instrumental in pain pathophysiology 34, 37.

Auxiliary proteins can modulate K_v_ function by affecting gating, expression levels and trafficking. For instance, K_v_β2 subunits enhance K_v_1 currents by promoting trafficking and membrane incorporation as well as inhibiting inactivation, whereas K_v_β1 subunits confer the opposite effects [127]. Genetic K_v_β2 deletion leads to defects in axonal targeting of K_v_1.1 and K_v_1.2, reduced after-hyperpolarization, and increased amygdala hyperexcitability associated with memory impairments [128]. In myelinated DRG neurons, K_v_β2.1 colocalizes with K_v_1.1 and K_v_1.2; however, the modest Kvβ2.1 reduction by injury (25%) argues against a significant role in K_v_1 dysfunction in chronic pain 25, 26. Nevertheless, targeting auxiliary subunits may be of therapeutic value; for example Kv1.1 ‘disinactivators’ reduce excitability by preventing K_v_1.1 inactivation by K_v_β1 [129].

Similarly, the localization of AMIGO (amphoterin-induced gene and ORF) dynamically follows that of K_v_2.1, and AMIGO increases K_v_2.1 conductance [130]. K_v_4 surface expression and gating can be enhanced by K_v_ channel interacting proteins (KChIPs), which respond to intracellular calcium fluxes during AP firing, and by dipeptidyl-peptidase-like proteins (DPPs) [131]. Interestingly, these auxiliary proteins often have distinctive distributions that may be relevant to pain processing. For instance, KChIP3 is abundant in medium-large DRG neurons, whereas DPP10 is restricted to small neurons [132]. Finally, K_v_7.2/K_v_7.3 can be modulated by MinK-related peptides (MiRPs, pluripotent proteins that also interact with K_v_2, K_v_3, and K_v_4), calmodulin, and A-kinase anchor proteins (AKAPs) [131]. The above description is by no means exhaustive; interested readers are referred towards excellent topical reviews [131].

Manipulation of K^+^ channel subunits with dominant contributions in neuron excitability is likely to play a key role in shaping future pain treatments. The development of novel technologies and increasing availability of structural information creates an optimistic outlook for pharmacological design of K^+^ channel modulators 108, 109, 110. In the next few years these advancements may be complemented by gene therapy strategies to introduce K^+^ channel copies at lesioned sites of the nervous system. Given the considerable convergence of pain mechanisms, it is plausible that synergistic treatments with K^+^ channel openers and other drugs (e.g., sodium or calcium channel blockers) can improve analgesic outcomes and/or circumvent side-effects by expanding the therapeutic window of present drugs to lower, more tolerable doses.
